# The Tomato Interspecific NB-LRR Gene Arsenal and Its Impact on Breeding Strategies

**DOI:** 10.3390/genes12020184

**Published:** 2021-01-27

**Authors:** Giuseppe Andolfo, Nunzio D’Agostino, Luigi Frusciante, Maria Raffaella Ercolano

**Affiliations:** Department of Agricultural Sciences, University of Naples Federico II, Via Università 100, 80055 Portici (NA), Italy; giuseppe.andolfo@unina.it (G.A.); nunzio.dagostino@unina.it (N.D.); fruscian@unina.it (L.F.)

**Keywords:** R-genes, gene clusters, NLR genes, evolutionary dynamics, genomic-driven breeding

## Abstract

Tomato (*Solanum lycopersicum* L.) is a model system for studying the molecular basis of resistance in plants. The investigation of evolutionary dynamics of tomato resistance (R)-loci provides unique opportunities for identifying factors that promote or constrain genome evolution. Nucleotide-binding domain and leucine-rich repeat (NB-LRR) receptors belong to one of the most plastic and diversified families. The vast amount of genomic data available for Solanaceae and wild tomato relatives provides unprecedented insights into the patterns and mechanisms of evolution of NB-LRR genes. Comparative analysis remarked a reshuffling of R-islands on chromosomes and a high degree of adaptive diversification in key R-loci induced by species-specific pathogen pressure. Unveiling NB-LRR natural variation in tomato and in other Solanaceae species offers the opportunity to effectively exploit genetic diversity in genomic-driven breeding programs with the aim of identifying and introducing new resistances in tomato cultivars. Within this motivating context, we reviewed the repertoire of NB-LRR genes available for tomato improvement with a special focus on signatures of adaptive processes. This issue is still relevant and not thoroughly investigated. We believe that the discovery of mechanisms involved in the generation of a gene with new resistance functions will bring great benefits to future breeding strategies.

## 1. Introduction

Tomato (*Solanum lycopersicum* L.) belongs to the large and diverse Solanaceae family, also referred to as nightshade. Species in the *Solanum* section *Lycopersicon* originated in the region that extends from Andean Highlands to the coast of Galapagos islands and includes the domesticated tomato and its 12 closest wild relatives (*S. arcanum*, *S. cheesmaniae*, *S. chilense*, *S. chmielewskii*, *S. corneliomulleri*, *S. galapagense*, *S. habrochaites*, *S. huaylasense*, *S. neorickii*, *S. pennellii*, *S. peruvianum* and *S. pimpinellifolium*) [1].

The economical and nutritional importance places tomato among the most widely studied crops, thus making it a model to understand the molecular processes related to plant–pathogen interaction [2]. A wide range of biotic stresses impairs tomato yield, most of which have been largely investigated to better understand which molecular mechanisms are activated during diseases [3].

The tomato genome sequence has been released over eight years ago [4], totally revolutionizing the pace of breeding activities. Scientists and breeders around the world actively use the genome to investigate the tomato “sequence space” with different goals. The first exhaustive annotation of tomato R-genes was released six years ago [5], and a tomato R-gene functional map was published immediately after [6].

Wild tomato species are characterized by a wide genetic variability as they occupy different habitats along a diversified climatic gradient [7]. By contrast, cultivated tomato has faced several bottlenecks during its domestication history; this led to a drastic reduction of its genetic diversity [8,9]. Therefore, it is necessary to recover the untapped variability of wild tomato relatives, as they represent the primary source of resistance for the cultivated tomato, being rich in genes conferring resistance to a large panel of pathogens [10]. The identification of suitable sources of new resistance is one of the most straightforward strategies for obtaining pathogen-resistant tomato varieties. Therefore, the identification and characterization of resistance (R)-genes in tomato wild relatives is definitely useful for both classical and innovative breeding strategies.

As the amount of sequenced genomes increases, comparative genomics is becoming an even more powerful method for identifying functionally important loci [11]. Comparative approaches across Solanaceae species have proved particularly useful for gaining new insights into the evolution and functional diversification of R-genes. Investigations on R-gene families in other Solanaceae species (potato, pepper, and eggplant) have helped to consolidate our knowledge on the processes that mediate disease resistance/tolerance within Solanaceae and, more generally, within plants [12].

Basically, to defend themselves, plants have developed a complex defense system to quickly recognize invading pathogens and transmit the message of attack [13,14]. The innate immunity system of plants has evolved in two recognition layers (PTI: PAMP (pathogen-associated molecular pattern)-triggered immunity and ETI: effector-triggered immunity). Plants’ own numerous non-self-recognition receptors are able to identify enemy molecules and induce a set of pathways and signaling cascades to repel attacks [15,16]. In ETI, immunity is mainly activated through the recognition of pathogen effectors via plant disease R-proteins. The major class of R-genes is represented by members of the nucleotide-binding site and leucine-rich repeat (NB-LRR or NLR) gene family [17]. The activation of NB-LRRs, during ETI response, induces programmed cell death, known as hypersensitive response (HR) [18,19]. Historically, NB-LRRs are divided into two subclasses, namely TIR-NB-LRR (TNL) and CC-NB-LRR (CNL) [20]. In addition to their role in pathogen recognition, some NB-LRR proteins contribute to signal transduction and/or amplification. Recent studies described a novel NB-LRR subclass, tagged as RPW8-NB-LRR (RNL, also known as helpers), whose protein members carry the RPW8 (resistance to powdery mildew 8) domain at the N terminal. RNLs mediate the immune response by interacting with NB-LRR “sensor” proteins involved in the detection of pathogens [21]. Additional NB-LRR helpers, which belong to a separate CNL subclass and exhibit high sequence similarity with NRC1 (NB-LRR protein required for HR-associated cell death 1), are required for cell death mediated by NB-LRR sensors [22]. NB-LRR helpers turned out to be essential in tomato, not only to support the activity of NB-LRR sensors, but also to counteract plant pathogens that evolve quickly, thus increasing the robustness of the innate immune system [23,24].

In this work, we present the tomato model system for studying the molecular basis of immunity activation in plant. The tomato NB-LRR repertoire was revised in the light of all the adaptive processes that have shaped it. The evolutionary dynamics of tomato R-loci were reviewed to reveal the main forces that shaped genome evolution. The information on R-genes gathered so far in major Solanaceae crops and wild tomato relatives set the stage to develop innovative genomic-driven breeding strategies, aimed to furnish novel resistance sources.

## 2. The Genome-Wide Arrangement of Tomato NB-LRR Genes

NB-LRR genes belong to a protein family with a very variable number of members among plants, ranging from about 50 to over 1000 [25]. A total of 294 NB-LRR genes were automatically identified and characterized by Andolfo et al. [5] and later revisited by Chandraprakash and Thomas [26] starting from the gene annotation released by the international Tomato Annotation Group (iTAG). However, that process failed to identify few genes, so the tomato NB-LRR complement was fully re-annotate by using the RenSeq method [3].

*S. lycopersicum* harbors approximately 320 NB-LRR-encoding genes arranged on all 12 chromosomes, whose genome-wide distribution is not random [3]. The largest number of NB-LRR genes is on chromosomes 4, 5, and 11 (~45%), whilst the smallest number is on chromosome 3 (9 genes), as found in other Solanaceae [12]. Some chromosomes predominantly host members of specific subclasses. Chromosomes 4 and 5 are particularly rich in CNLs. The largest number of TNLs (43%) is on chromosome 1, while chromosomes 3, 6, and 10 do not have them. Full-length RNL genes are only on chromosomes 2 and 4 [25], and are orthologous of the *Arabidopsis thaliana* NRG1 (N requirement gene 1) and *Nicotiana benthamiana* ADR1 (Activated Disease Resistance 1) R-gene helpers [27,28]; while tomato NRC1-homologs were located on chromosomes 2 and 10 [22].

The tomato NB-LRR repertoire includes also about 100 proteins that lack the full complement of domains that characterize genes within the NB-LRR subclasses. These include 14 CC-NB (CN) and three TIR-NBS (TN) proteins that have no LRR domain [3]. The majority of incomplete NB-LRR genes (~80%) only own a single domain and their function is still unknown, although it has been speculated they could act as adaptors or regulators of NB-LRR proteins with proven resistance activity [29]. In tomato the number of incomplete NB-LRR genes with evidence of expression is higher than observed in other plant species [3].

Generally, NB-LRR-encoding genes are clustered, as result of both segmental and tandem duplications [12,16]. Over 65% of tomato NB-LRRs are gathered in small genomic regions spanning 200 kb or less. One third of NB-LRRs (107 genes) are concentrated in 20 clusters [12]. This specific organization reflects genomic hotspots for diversification. The largest tomato cluster harbors 14 CNL genes in a region of ~110-kb in size on the short arm of chromosome 4. All members of this cluster share high sequence similarity with the wild potato derived R-genes R2, Rpi-blb3, and Rpi-abpt [30]. It is likely that functional R-genes, which have not yet been identified, are in this rapidly evolving cluster. 

In tomato, several NB-LRR clusters comprised genes encoding proteins with high similarity to known and well-characterized R-genes [5]. Deciphering the evolutionary history of a gene cluster is essential not only to discover the functional specificity of each cluster-related allele, but also to provide insights into genome diversification by species. The increasing amount of cloned R-genes (Table 1) over the last two decades paved the way for the investigation of the evolutionary dynamics that generate novel resistance, which arise to match the changing patterns of pathogen virulence [31].

Tomato NB-LRR loci are preferentially located in recombination hotspots, where meiotic crossovers are more frequent. Interestingly, all tomato cloned NB-LRR resistance genes, but not *Tm2*^2^ and *Prf* conferring a fairly durable resistance [32,33], lie in regions exhibiting high/medium rates of recombination. The choice to retain the resistance loci into hot or cold recombination regions may reflect a different evolutionary state of pathogen–plant interactions. Recombination may be favorable in gene families controlling resistance to highly variable pathogens but unfavorable in families that control resistance to pathogens with low genetic plasticity [34]. The knowledge on the potential effects of crossover frequency on the tomato genome is prerequisite to predict R-gene haplotypes emerging following hybridization events.

**Figure 1:**
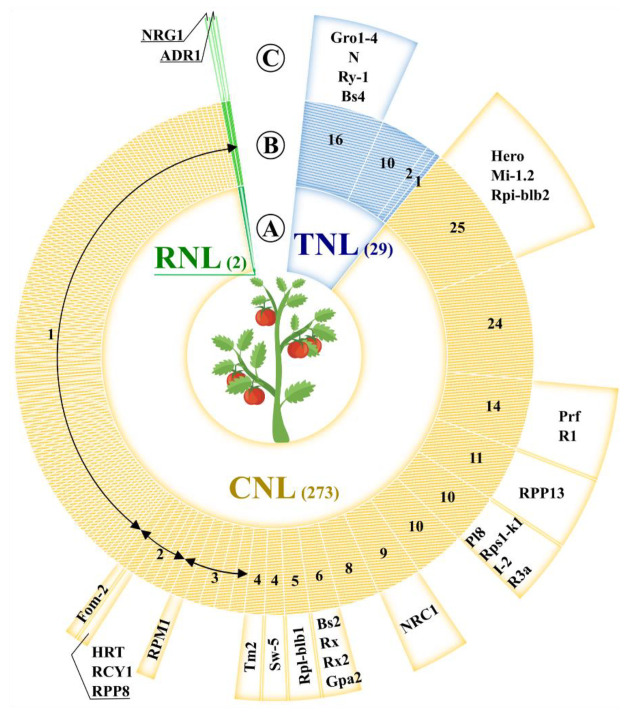
The tomato defense arsenal. (**A**) The full R-gene repertoire was displayed with respect to the NB-LRR subclasses (TNL in blue; CNL in orange and RNL in green). The total number of CNLs, TNLs, and RNLs was shown in brackets. (**B**) The NB-LRR paralogs identified by OrthoMCL with default settings were grouped (annular segments) and the amount of members in each group was specified. (**C**) For each group of paralogs, the well-characterized R-gene homolog was indicated (Table S1), when available.

The recombination density can increase the drift of genes in large clusters, such as I2 and Mi, which are in adaptive evolutionary state and have shown a frequency of recombination that is twice the average [35]. These two super-clusters, which include the R-genes *Mi* and *I-2* [36,37], conferring resistance to *Meloidogyne incognita* and *Fusarium oxysporum* f. sp. *Lycopersici* respectively, have been extensively studied. The I2 super-cluster comprises seven NB-LRR genes gathered in a region of 390 kb on chromosome 11. The *I-2* homolog genes were grouped in two sub-clusters of 54 kb and 28 kb in size. Similarly, six CNLs are in the Mi super-cluster, which is split into two sub-groups on chromosome 6. A region of approximately 400 kb was involved in the intra-chromosomal duplications and generated the Mi super-cluster [5]. In several plant genomes, the NB-LRR-encoding genes have been amplified, thus resulting in species-specific sub-families [16]. The diversification of tomato R-gene arsenal was mediated by duplication events of distinct NB-LRR paralogs (Figure 1). Forty-five out of ~320 NB-LRR sequences in tomato are more similar to each other than to any other non-Solanaceae sequences. Indeed, large gene expansions (more than 15 copies), involving unknown NB-LRRs—as well as *Prf*/*R1*, *Hero*/*Mi 1.2*, *Gro1-4*/*N* members—were observed.

## 3. Resistance Sources in Wild Tomato Relatives

Wild tomato species are characterized by a wide genetic variability and phenotypic diversity. As they harbor genes involved in resistance to diseases and tolerance to abiotic stresses, they have long been used as donor of genes/alleles, thus playing a key role in the improvement of cultivated varieties. Indeed, domestication, early breeding and artificial selection led to the loss of some important features in cultivated tomato, including resistances to diseases and pests. Exploring natural biodiversity in wild tomato relatives is key to discovering new genetic sources of resistance.

The inclination of plant species to survive over evolutionary time depends on their ability to usefully generate and maintain diversity at resistance loci. Over the past decades, a continue effort to identify novel resistance genes, including NB-LRRs, was conducted in wild tomato species from diverse habitats. Comparative genomics revealed the huge potential of wild R-gene diversity. NB-LRR genes in *S. pimpinellifolium*, *S. pennellii*, and *S. chilense* are mainly located in *S. lycopersicum* corresponding loci. However, gene repertories reveal important amplification and contraction in specific NB-LRR subfamilies [49,50,51]. Natural variation that occurs in several wild tomato species show that the evolutionary history is characterized by lineage sorting of the polymorphisms and that the generation of new variants events have shaped R-gene diversity after speciation. Patterns of evolution from wild to cultivated species were largely unexplored, but useful insights have recently been gained from genomic studies [10,52,53]. Nucleotide variation in specific positions have dramatic effects on the intra- or inter-molecular activity of R-proteins and, hence, in resistance response to pathogens and pests [54]. In addition, signatures of adaptation to different habitats are more marked in sensor-NBS-LRRs than in helper genes [10]. The centrality of NB-LRRs in gene networks does not impair their evolution, and new mutations in key genes (hubs) of the network are important for R-gene adaptation during colonization of different habitats. Recently, NB-LRR genes of 15 wild tomato accessions belonging to 5 species (*S. cheesmaniae*, *S. chmielewskii*, *S. galapagense*, *S. neorickii* and *S. pimpinellifolium*) were identified by combining RenSeq with single-molecule real time (SMRT) sequencing [55]. Seong et al. [55] reported that the amount of NB-LRR genes ranged from 332 to 264, in *S. galapagense* and *S. neorickii* respectively. Comparative genomic analysis revealed how evolution has reshaped R-gene clusters in close wild tomato relatives. The large helper-sensor networks have been shown to be one of the major drivers of NB-LRR evolution in tomato, as they are highly variable in number in different wild tomato species and display changing evolutionary patterns determined by the peculiar interaction with pathogens [55]. All these findings enhance our understanding of the dynamic evolution of NB-LRRs and provide insights and a solid foundation for future breeding and molecular engineering for disease resistance.

## 4. Evolution of R-Type Defense Genes within Solanaceae

Distinct patterns of evolution shaped the repertoire of NB-LRR-encoding genes of Solanaceae species [56]. A wide variation in the arrangement of protein domains was found [11]. A ‘continuous expansion’ pattern was observed in potato and an overall ‘shrinking’ pattern in pepper. By contrast, tomato NB-LRR-encoding genes exhibit an ‘expansion followed by contraction’ pattern, as it lost many genes after the divergence of potato and tomato. The evolutionary history of NB-LRR loci revealed several species-specific gene expansions in Solanaceae. The comparison between tomato, eggplant and pepper proteomes evidenced orthologous proteins across the three cultivated Solanaceae species [12]. An expansion of the Mi1.2 locus (R-gene against *Meloidogyne incognita*) and of I2 locus (resistance to *Fusarium oxysporum* f. sp. *Lycopersici*) was identified in four Solanaceae [11]. Gene expansions, involving genes homologous to Bs2 (resistance to *Xanthomonas campestris* pv. *vescicatoria* in pepper), *Tm2*^2^ and Sw5 (resistance to ToMV and TSWV in tomato) and R2 (resistance to *Phytophthora infestans* in potato), were found in pepper, potato and tomato respectively. An increasing number of Ry1 and N members was observed in eggplant (chromosomes 5 and 11), and potato (chromosome 9) [11]. Furthermore, some loci were scattered on different chromosomes in other species [57]. The Gpa2 locus on tomato chromosome 12 is orthologous to two loci on pepper chromosome 9. Clusters on tomato chromosome 9, including the genes Sw5 and *Tm2*^2^, have correspondence to R-islands on pepper chromosome 3 [58,59]. Syntenic regions in eggplant and pepper are larger than the corresponding ones in tomato, possibly due to the difference in genome size of the species under investigation [4,11,60].

Comparative analysis among selected NB-LRR loci showed a large number of genome rearrangements (gene duplications/losses, genome reshuffling, and transposable element insertions). In particular, pepper showed a massive insertion of transposable elements in NB-LRR-encoding genes located on specific chromosomes [61]. R-gene architecture seems to be modified by the interplay of large-scale gene organization, that determines global conservation in the order of loci, and extensive local genome rearrangements mediated by tandem duplication, transposons, and other reshuffling elements [62]. Extant local arrangements in R-loci suggest that adaptive diversification is induced by species-specific pathogen pressure. In general, larger clusters are generated to promote the diversification of crucial resistance loci in certain species [12].

Solanaceae genome architecture has evolved by preserving highly active R-islands, where local genomic variability is regulated in a species-specific manner. Indeed, recombination, chromosome breaking points, and other elements (e.g., transposons) that locally promote R-block rearrangement facilitated R-gene diversification [11]. NB-LRR copy number variation likely results from the need to maintain a diverse array of genes, that has been generated following events of duplication and divergence, and to retain advantageous resistance specificities. The quality and intensity of pathogen virulence together with the genome plasticity of plants define the direction and magnitude of lineage-specific R-gene expansions. From a genome-wide perspective, the parallel evolution of species within the Solanaceae family allowed to design species-specific defense arsenals by combining the retention of strategic duplications occurred millions of years ago [63] with high dynamic selection at given R-loci. 

## 5. Genomic-Driven Breeding for Developing New Resistant Tomato Varieties

**Figure 2:**
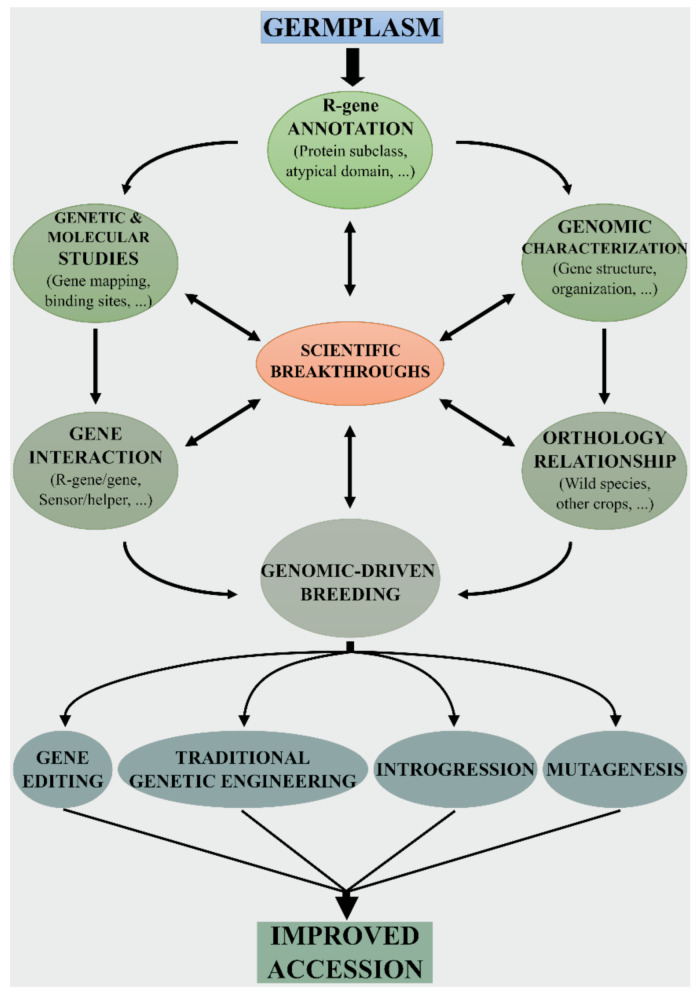
Genomic-driven breeding workflow for disease resistance. The identification, annotation, and characterization of NB-LRR genes are essential to investigate the extent of variability and compare the defense arsenals typical of each tomato/Solanaceae species. Information on gene–gene interaction provides additional evidence on the role and function of NB-LRRs. The different analytical approaches based on the most cutting-edge technologies must be combined to gain valuable knowledge on R-genes. The expanded knowledgebase is then used to apply the most appropriate genome-driven breeding technique to improve tomato disease resistance.

The growing body of knowledge on the Solanaceae genomes will undoubtedly expedite the transfer of beneficial traits into tomato (Figure 2). A large number of R genes (including NB-LRR) have been introgressed from tomato wild relatives [64,65,66]. For example, sources of resistance against root-knot nematodes, aphids, whiteflies, viruses (TMV, TYLC, TSWV), and fungi were found in *S. arcanum*, *S. habrochaites*, *S. pennelli* and *S. peruvianum*, (Table 1). In addition, several genes derived for other Solanaceae species extended the tomato gene pool via transformation [67,68,69].

Traditional breeding based on “introgressomics” [70] greatly promoted the transfer of wild resistance genes in tomato. After the selection of the most eligible wild species and its hybridization with the cultivated species, the resistance traits are introgressed into the cultivated background after several generations of backcrossing. However, linkage drag (i.e., the undesirable effects of genes linked to the gene to be introgressed) is often associated with traditional introgression. As an example, the *S. peruvianum* introgression carrying the tomato mosaic virus (ToMV) resistance gene *Tm2*^2^ can cover up to 79% of chromosome 9 in modern tomato varieties [71]. Therefore, it is highly desirable to establish reliable and more and more precise methods that can broaden genetic diversity through the introgression of alleles from wild relatives. The generation of new combinations of resistance alleles should be assisted by genomic-driven tomato breeding to minimize unwanted traits.

Genome editing technologies can make the “rewilding” process more easy [72], as they allow for the precise introduction of desirable genes/alleles from related wild relatives into elite cultivars (Figure 2). Indeed, plant disease resistance can be enhanced by targeting different genes of the plant defense machinery [73]. To edit NB-LRR genes it would be necessary modifying single nucleotides via “base-editing” and/or via homology-directed repair (HDR)-mediated base substitution [74]. In this case, homologous sequences serve as donors to repair site-specific double-strand DNA breaks caused by site-directed nucleases. Indeed, it is known that a few amino acid binding sites in different domains of NB-LRR proteins are required for pathogen recognition. Knowledge on the pathogen recognition sites of a particular NB-LRR receptor can be used to improve homologous NB-LRR in other accessions/species. As an example, Giannakopoulou et al. [75] assessed the degree of response of I2 random mutants to the *Phytophthora infestans* effector AVR3a. The mutant I2(I141N) conferred partial resistance to *P. infestans* and had a wider response spectrum to *F. oxysporum* f. sp. *lycopersici* effectors. Similarly, Segretin et al. [76] performed a gain-of-function random mutagenesis screen of the potato NB-LRR immune receptor R3a expanding its response to the *P. infestans* effector AVR3a. Remarkably, they found that the N336Y mutation conferred response also to the effector protein AVR3a4 from *P. capsici*.

To the best of our knowledge, however, no ‘base editing’ experiments or HDR-mediated base substitution has been attempted yet for NB-LRR genes in tomato or other Solanaceae. Genome editing technologies can also be used to edit or insert specific cis-regulatory elements into promoter regions, alter the epigenetic status [77] and to target miRNAs controlling the expression of NB-LRR genes with both cis- and trans-regulatory effects [78,79]. Indeed, it was observed that the silencing of miR482b in tomato plants promote the expression of several NBS–LRR receptors, thus enhancing resistance to *P. infestans* [80]. Transcriptional suppression may act as buffer for R-genes, thus reducing constraints on R-gene sequences [81].

Recent studies have shown that immune responses are mediated by complex and knotty networks of genes [23]. In some cases, the involvement of additional loci can make difficult the transfer of resistance traits in a new genetic background. For an optimal response to pathogens, it would therefore be appropriate to preserve almost entirely the wild NB-LRR network so that the new developed varieties will have greater ability to react to stresses. As a consequence, a viable and alternative solution for designing resistant tomato varieties is the de novo domestication of wild species [82,83]. With a particular focus on the concept of signaling by cooperative assembly formation, it would be desirable to exploit the wild genetic background by going to edit only key domestication/improvement genes [83,84,85]. Indeed, the expansion of the pathogen sensor functionality can require to bring together a large number of actors that can be activated in a proximity-based manner resulting in a higher-magnitude signal [86]. Understanding the evolution, assembly, and regulation of tomato immune receptor circuits is crucial to delivering fine-tuned tomato resistant varieties.

## 6. Remarks

NB-LRR natural variation in tomato wild relatives and in other Solanaceae species offers the opportunity to effectively exploit genetic diversity with the aim of developing new resistant cultivars. Knowledge on the NB-LRR gene repertoire, chromosome organization, spatio–temporal regulation and mechanisms involved in the birth of a gene with new resistance functions will great benefit future breeding strategies. Genomic recombination data can better direct the use of resistance genes in crossing schemes even though it is still difficult to combine tightly linked resistance genes from different sources. Genome editing technologies can be crucial for determining the functional significance of cataloged elements and for transferring desirable genes/alleles from wild tomato relatives into cultivated varieties. These gene editing technologies are A viable and alternative solution for designing resistant tomato varieties with an improved ability to react to stresses is re-modulate NB-LRR networks.

## Figures and Tables

**Table 1:** List of cloned NB-LRR resistance genes in tomato and its wild relatives. Gene name, protein class, chromosome number and source of resistance for each R-gene were reported.

Gene Name	Protein Class	Chromosome	Pathogen/Insect	Source of Resistance	Reference
*Bs4*	TNL	5	*Xanthomonas campestris* pv. *vesicatoria*	*S. lycopersicum*	Ballvora et al. [38]
*Hero*	CNL	4	*Globodera rostochiensis*	*S. pimpinellifolium*	Ernst et al. [39]
*I-2*	CNL	11	*Fusarium oxysporum* f. sp. *Lycopersici*	*S. pimpinellifolium*	Simons et al. [37]
*Mi-1.2*	CNL	6	*Melaydogyne* spp.; *Macrosiphum euphorbiae*; *Bemisia tabaci*	*S. peruvianum*	Rossi et al. [36] Mahfouze et al. [40]
*Mi-9*	CNL	6	*Meloidogyne* spp.	*S. arcanum*	Jablonska et al. [41]
*Prf*	CNL	5	*Pseudomonas syringae*	*S. pimpinellifolium*	Salmeron et al. [42]
*Ph-3*	CNL	9	*Phitophthora infestans*	*S. pimpinellifolium*	Zhang et al. [43]
*Sw-5*	CNL	9	Tomato spotted wilt virus	*S. peruvianum*	Brommonschenkel et al. [44]
*Tm-1*	CNL	2	Tomato mosaic virus	*S. habrochaites*	Ishibashi et al. [45]
*Tm-2*	CNL	9	Tobacco mosaic virus	*S. habrochaites*	Lanfermeijer et al. [46]
*Tm-2a*	CNL	9	Tobacco mosaic virus	*S. peruvianum*	Lanfermeijer et al. [47]
*Ty 2*	CNL	11	Tomato leaf curly yellow virus	*S. habrochaites*	Yang et al. [48]

## Supplementary Materials

The following are available online at https://www.mdpi.com/2073-4425/12/2/184/s1, Table S1: Reference NB-LRR genes for comparative purposes. List of 29 cloned and characterized plant NB-LRR genes used as reference.
