# Supplementary material for: The Tomato Interspecific NB-LRR Gene Arsenal and Its Impact on Breeding Strategies
Source: Genes (Basel). 2021 Jan 27;12(2):184. doi: 10.3390/genes12020184 (PMC7911644; doi:10.3390/genes12020184)
Supplement: Supplementary file 1 [file genes-12-00184-s001.pdf]

Additional File 1

Reference NB-LRR genes for comparative propose.

Gene name	Reference species	Protein subclass	Disease
**ADR1**	*Arabidopsis thaliana*	RNL	Powdery mildew
**Bs2**	*Capsicum chacoense*	CNL	Bacterial spot disease
**Bs4**	*Solanum lycopersicum*	TNL	Bacterial spot disease
**Fom-2**	*Cucumis melo*	CNL	Fusarium wilt
**Gpa2**	*Solanum tuberosum*	CNL	Yellow potato cyst nematode
**Gro1-4**	*Solanum tuberosum*	TNL	Late blight
**Hero**	*Solanum lycopersicum*	CNL	Yellow potato cyst nematode
**HRT**	*Arabidopsis thaliana*	CNL	TCV
**I-2**	*Solanum lycopersicum*	CNL	Fusarium wilt
**Mi-1.2**	*Solanum lycopersicum*	CNL	Root-knot nematode
**N**	*Nicotiana glutinosa*	TNL	TMV
***NRC1**	*Nicotiana benthamiana*	CNL	-
**NRG1**	*Nicotiana benthamiana*	RNL	TMV
**Pl8**	*Helianthus annuus*	CNL	Downy mildew
**Prf**	*Solanum lycopersicum*	CNL	Bacterial Speck
**R1**	*Solanum demissum*	CNL	Late blight
**R3a**	*Solanum demissum*	CNL	Late blight
**RCY1**	*Arabidopsis thaliana*	CNL	CMV
**Rpi-blb1**	*Solanum bulbocastanum*	CNL	Late blight
**Rpi-blb2**	*Solanum bulbocastanum*	CNL	Late blight
**RPM1**	*Arabidopsis thaliana*	CNL	Bacterial blight
**RPP13**	*Arabidopsis thaliana*	CNL	Downy mildew
**RPP8**	*Arabidopsis thaliana*	CNL	Downy mildew
**Rps1-k1**	*Glycine max*	CNL	Root and stem rot disease
**Rx**	*Solanum tuberosum*	CNL	PVX
**Rx2**	*Solanum acaule*	CNL	PVX
**Ry-1**	*Solanum tuberosum*	TNL	PVY
**Sw-5**	*Solanum peruvianum*	CNL	Tomato spotted wilt
**Tm2**	*Solanum lycopersicum*	CNL	ToMV

*Required for cell death mediated by NB-LRR sensors.

**Phatogen/Insect**
Erysiphe cichoracearum
Xanthomonas campestris pv. vesicatoria
Xanthomonas campestris pv. vesicatoria
Fusarium oxysporum f.sp. melonis
Globodera
Phytophthora infestans
Globodera
Turnip crinkle virus
Fusarium oxysporum
Meloidogyne, Paratrichodorus minor
Tobacco mosaic virus
-
Tobacco mosaic virus
Plasmopara halstedii
Pseudomonas syringae pv. tomato
Phytophthora infestans
Phytophthora infestans
Cucumber mosaic virus
Phytophthora infestans
Phytophthora infestans
Pseudomonas syringae
Hyaloperonospora parasitica
Hyaloperonospora parasitica
Pytophthora sojae
Potato virus X
Potato virus X
Potato virus Y
Tomato spotted wilt virus
Tomato mosaic virus
